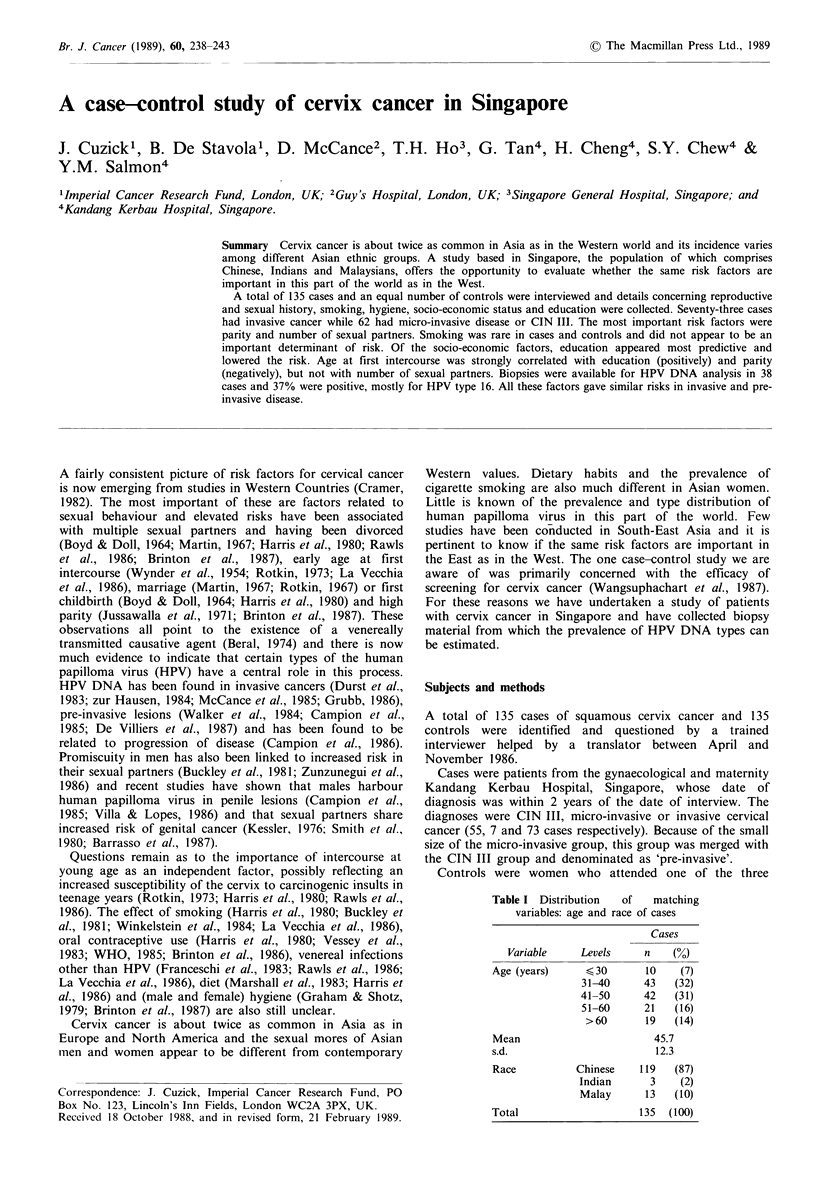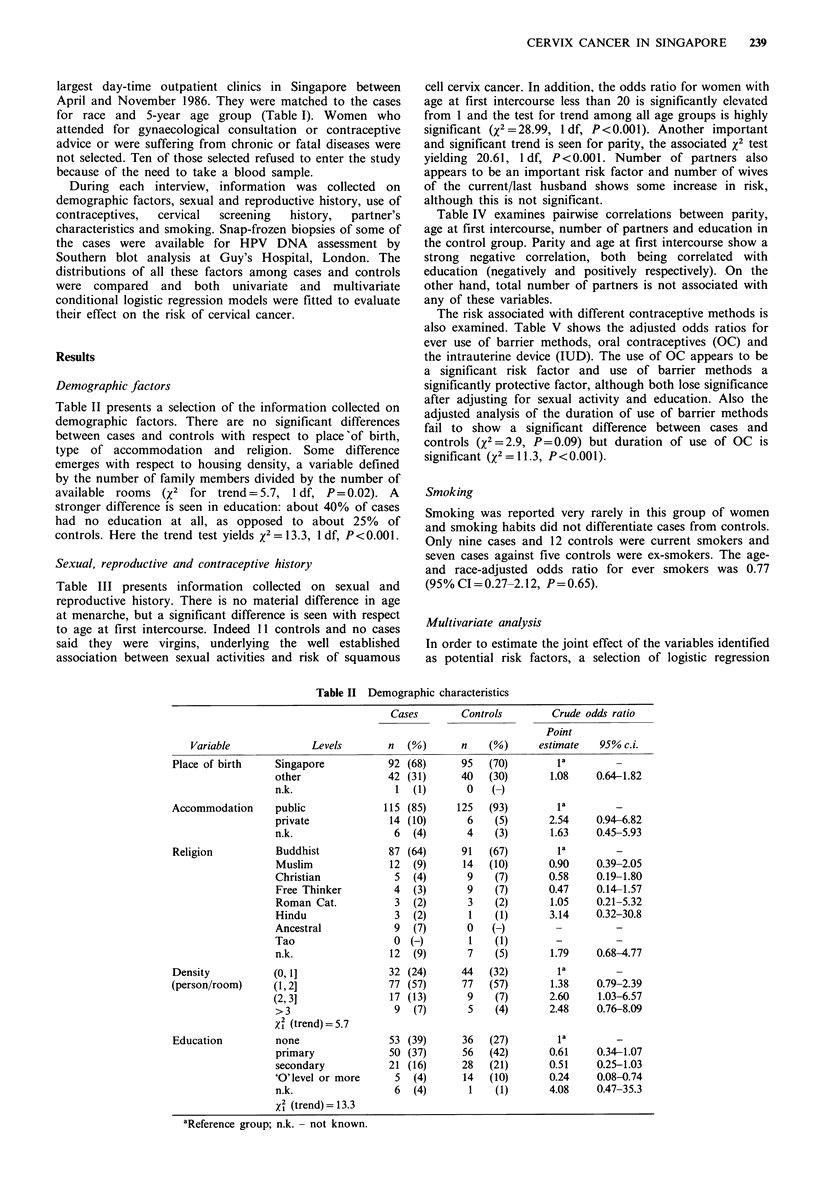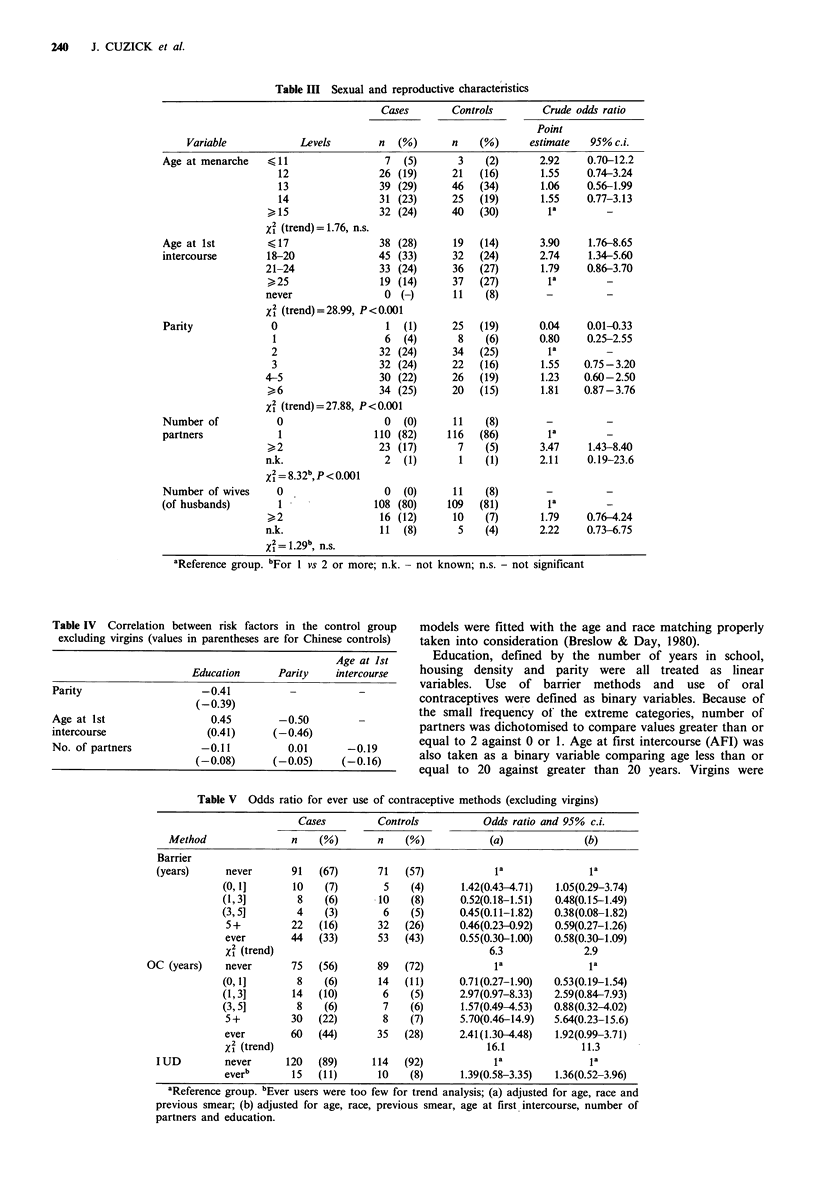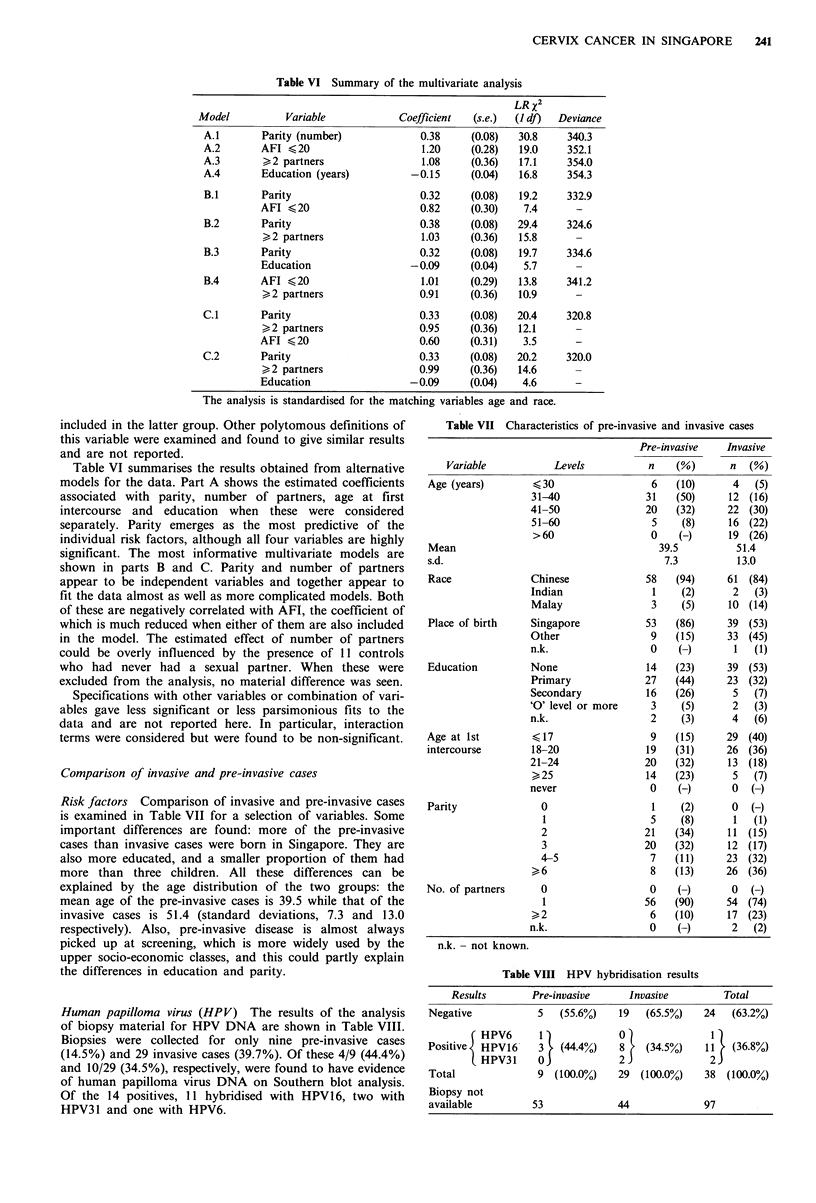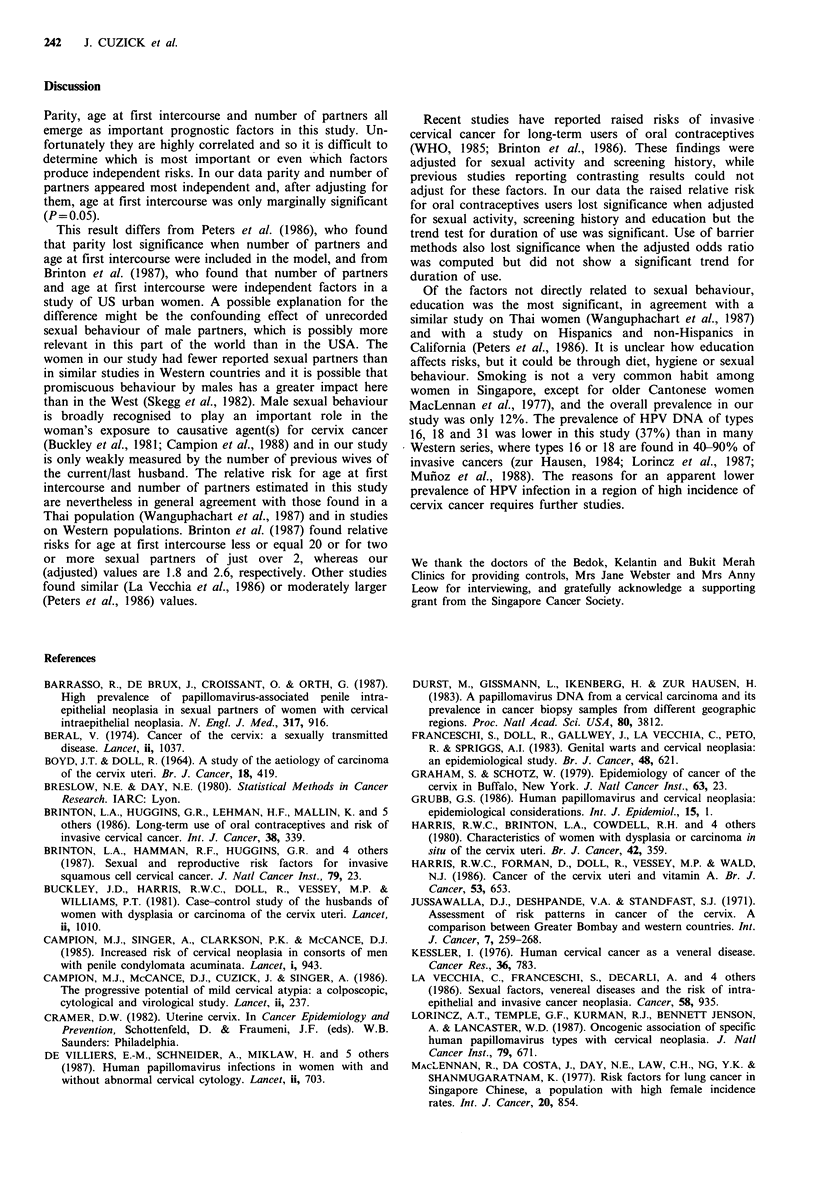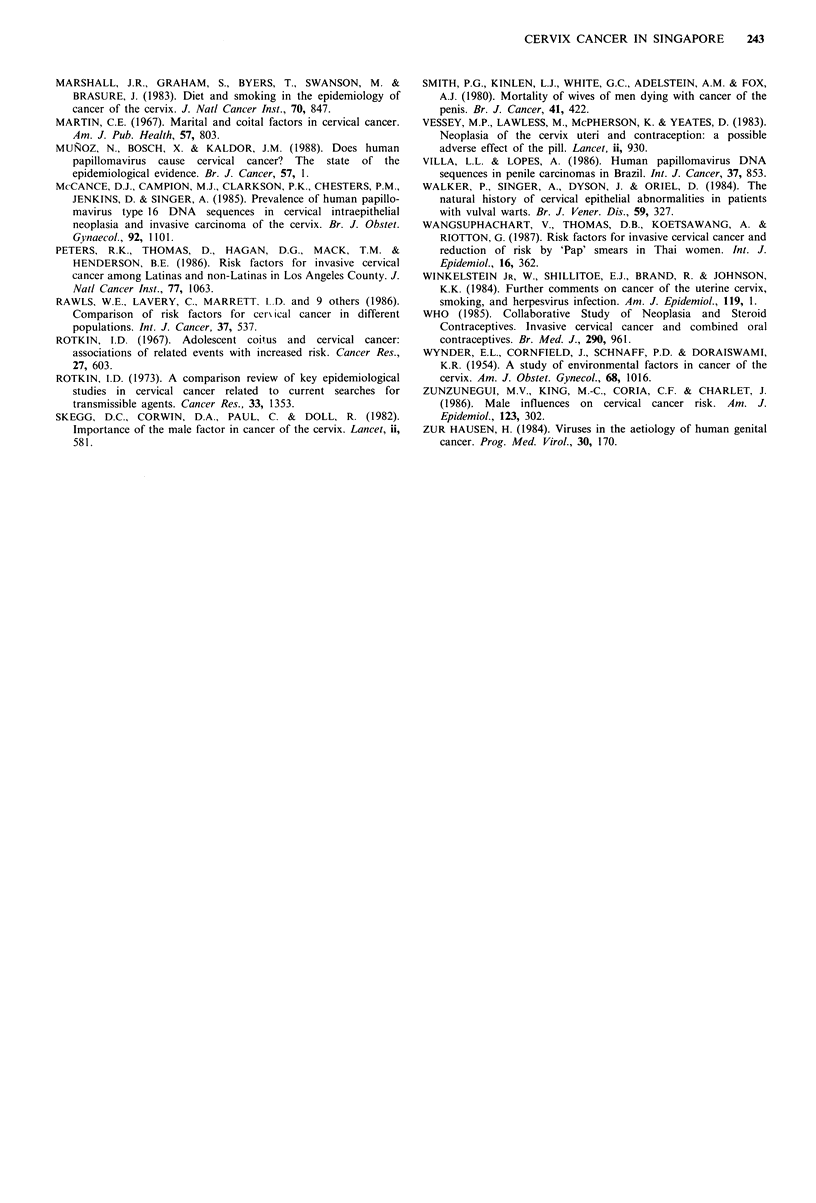# A case-control study of cervix cancer in Singapore.

**DOI:** 10.1038/bjc.1989.261

**Published:** 1989-08

**Authors:** J. Cuzick, B. De Stavola, D. McCance, T. H. Ho, G. Tan, H. Cheng, S. Y. Chew, Y. M. Salmon

**Affiliations:** Imperial Cancer Research Fund, London, UK.

## Abstract

Cervix cancer is about twice as common in Asia as in the Western world and its incidence varies among different Asian ethnic groups. A study based in Singapore, the population of which comprises Chinese, Indians and Malaysians, offers the opportunity to evaluate whether the same risk factors are important in this part of the world as in the West. A total of 135 cases and an equal number of controls were interviewed and details concerning reproductive and sexual history, smoking, hygiene, socio-economic status and education were collected. Seventy-three cases had invasive cancer while 62 had micro-invasive disease or CIN III. The most important risk factors were parity and number of sexual partners. Smoking was rare in cases and controls and did not appear to be an important determinant of risk. Of the socio-economic factors, education appeared most predictive and lowered the risk. Age at first intercourse was strongly correlated with education (positively) and parity (negatively), but not with number of sexual partners. Biopsies were available for HPV DNA analysis in 38 cases and 37% were positive, mostly for HPV type 16. All these factors gave similar risks in invasive and preinvasive disease.


Br. J. Cancer (1989), 60, 238-243 © The Macmillan Press Ltd., 1989

### A case–control study of cervix cancer in Singapore

J. Cuzick[1], B. De Stavola[1], D. McCance[2], T.H. Ho[3], G. Tan[4], H. Cheng[4], S.Y. Chew[4] & Y.M. Salmon[4]

[1]*Imperial Cancer Research Fund, London, UK;* [2]*Guy's Hospital, London, UK;* [3]*Singapore General Hospital, Singapore; and* [4]*Kandang Kerbau Hospital, Singapore.*

**Summary** Cervix cancer is about twice as common in Asia as in the Western world and its incidence varies among different Asian ethnic groups. A study based in Singapore, the population of which comprises Chinese, Indians and Malaysians, offers the opportunity to evaluate whether the same risk factors are important in this part of the world as in the West.

A total of 135 cases and an equal number of controls were interviewed and details concerning reproductive and sexual history, smoking, hygiene, socio-economic status and education were collected. Seventy-three cases had invasive cancer while 62 had micro-invasive disease or CIN III. The most important risk factors were parity and number of sexual partners. Smoking was rare in cases and controls and did not appear to be an important determinant of risk. Of the socio-economic factors, education appeared most predictive and lowered the risk. Age at first intercourse was strongly correlated with education (positively) and parity (negatively), but not with number of sexual partners. Biopsies were available for HPV DNA analysis in 38 cases and 37% were positive, mostly for HPV type 16. All these factors gave similar risks in invasive and pre-invasive disease.

A fairly consistent picture of risk factors for cervical cancer is now emerging from studies in Western Countries (Cramer, 1982). The most important of these are factors related to sexual behaviour and elevated risks have been associated with multiple sexual partners and having been divorced (Boyd & Doll, 1964; Martin, 1967; Harris *et al.*, 1980; Rawls *et al.*, 1986; Brinton *et al.*, 1987), early age at first intercourse (Wynder *et al.*, 1954; Rotkin, 1973; La Vecchia *et al.*, 1986), marriage (Martin, 1967; Rotkin, 1967) or first childbirth (Boyd & Doll, 1964; Harris *et al.*, 1980) and high parity (Jussawalla *et al.*, 1971; Brinton *et al.*, 1987). These observations all point to the existence of a venereally transmitted causative agent (Beral, 1974) and there is now much evidence to indicate that certain types of the human papilloma virus (HPV) have a central role in this process. HPV DNA has been found in invasive cancers (Durst *et al.*, 1983; zur Hausen, 1984; McCance *et al.*, 1985; Grubb, 1986), pre-invasive lesions (Walker *et al.*, 1984; Campion *et al.*, 1985; De Villiers *et al.*, 1987) and has been found to be related to progression of disease (Campion *et al.*, 1986). Promiscuity in men has also been linked to increased risk in their sexual partners (Buckley *et al.*, 1981; Zunzunegui *et al.*, 1986) and recent studies have shown that males harbour human papilloma virus in penile lesions (Campion *et al.*, 1985; Villa & Lopes, 1986) and that sexual partners share increased risk of genital cancer (Kessler, 1976; Smith *et al.*, 1980; Barrasso *et al.*, 1987).

Questions remain as to the importance of intercourse at young age as an independent factor, possibly reflecting an increased susceptibility of the cervix to carcinogenic insults in teenage years (Rotkin, 1973; Harris *et al.*, 1980; Rawls *et al.*, 1986). The effect of smoking (Harris *et al.*, 1980; Buckley *et al.*, 1981; Winkelstein *et al.*, 1984; La Vecchia *et al.*, 1986), oral contraceptive use (Harris *et al.*, 1980; Vessey *et al.*, 1983; WHO, 1985; Brinton *et al.*, 1986), venereal infections other than HPV (Franceschi *et al.*, 1983; Rawls *et al.*, 1986; La Vecchia *et al.*, 1986), diet (Marshall *et al.*, 1983; Harris *et al.*, 1986) and (male and female) hygiene (Graham & Shotz, 1979; Brinton *et al.*, 1987) are also still unclear.

Cervix cancer is about twice as common in Asia as in Europe and North America and the sexual mores of Asian men and women appear to be different from contemporary Western values. Dietary habits and the prevalence of cigarette smoking are also much different in Asian women. Little is known of the prevalence and type distribution of human papilloma virus in this part of the world. Few studies have been conducted in South-East Asia and it is pertinent to know if the same risk factors are important in the East as in the West. The one case–control study we are aware of was primarily concerned with the efficacy of screening for cervix cancer (Wangsuphachart *et al.*, 1987). For these reasons we have undertaken a study of patients with cervix cancer in Singapore and have collected biopsy material from which the prevalence of HPV DNA types can be estimated.

### Subjects and methods

A total of 135 cases of squamous cervix cancer and 135 controls were identified and questioned by a trained interviewer helped by a translator between April and November 1986.

Cases were patients from the gynaecological and maternity Kandang Kerbau Hospital, Singapore, whose date of diagnosis was within 2 years of the date of interview. The diagnoses were CIN III, micro-invasive or invasive cervical cancer (55, 7 and 73 cases respectively). Because of the small size of the micro-invasive group, this group was merged with the CIN III group and denominated as 'pre-invasive'.

Controls were women who attended one of the three

**Table I** Distribution of matching variables: age and race of cases

| Variable | Levels | Cases n | (%) |
|---|---|---|---|
| Age (years) | ≤30 | 10 | (7) |
| | 31–40 | 43 | (32) |
| | 41–50 | 42 | (31) |
| | 51–60 | 21 | (16) |
| | >60 | 19 | (14) |
| Mean | | 45.7 | |
| s.d. | | 12.3 | |
| Race | Chinese | 119 | (87) |
| | Indian | 3 | (2) |
| | Malay | 13 | (10) |
| Total | | 135 | (100) |

Correspondence: J. Cuzick, Imperial Cancer Research Fund, PO Box No. 123, Lincoln's Inn Fields, London WC2A 3PX, UK.
Received 18 October 1988, and in revised form, 21 February 1989.

largest day-time outpatient clinics in Singapore between April and November 1986. They were matched to the cases for race and 5-year age group (Table I). Women who attended for gynaecological consultation or contraceptive advice or were suffering from chronic or fatal diseases were not selected. Ten of those selected refused to enter the study because of the need to take a blood sample.

During each interview, information was collected on demographic factors, sexual and reproductive history, use of contraceptives, cervical screening history, partner's characteristics and smoking. Snap-frozen biopsies of some of the cases were available for HPV DNA assessment by Southern blot analysis at Guy's Hospital, London. The distributions of all these factors among cases and controls were compared and both univariate and multivariate conditional logistic regression models were fitted to evaluate their effect on the risk of cervical cancer.

### Results

### *Demographic factors*

Table II presents a selection of the information collected on demographic factors. There are no significant differences between cases and controls with respect to place of birth, type of accommodation and religion. Some difference emerges with respect to housing density, a variable defined by the number of family members divided by the number of available rooms ($\chi^2$ for trend = 5.7, 1 df, $P = 0.02$). A stronger difference is seen in education: about 40% of cases had no education at all, as opposed to about 25% of controls. Here the trend test yields $\chi^2 = 13.3$, 1 df, $P < 0.001$.

### *Sexual, reproductive and contraceptive history*

Table III presents information collected on sexual and reproductive history. There is no material difference in age at menarche, but a significant difference is seen with respect to age at first intercourse. Indeed 11 controls and no cases said they were virgins, underlying the well established association between sexual activities and risk of squamous cell cervix cancer. In addition, the odds ratio for women with age at first intercourse less than 20 is significantly elevated from 1 and the test for trend among all age groups is highly significant ($\chi^2 = 28.99$, 1 df, $P < 0.001$). Another important and significant trend is seen for parity, the associated $\chi^2$ test yielding 20.61, 1 df, $P < 0.001$. Number of partners also appears to be an important risk factor and number of wives of the current/last husband shows some increase in risk, although this is not significant.

Table IV examines pairwise correlations between parity, age at first intercourse, number of partners and education in the control group. Parity and age at first intercourse show a strong negative correlation, both being correlated with education (negatively and positively respectively). On the other hand, total number of partners is not associated with any of these variables.

The risk associated with different contraceptive methods is also examined. Table V shows the adjusted odds ratios for ever use of barrier methods, oral contraceptives (OC) and the intrauterine device (IUD). The use of OC appears to be a significant risk factor and use of barrier methods a significantly protective factor, although both lose significance after adjusting for sexual activity and education. Also the adjusted analysis of the duration of use of barrier methods fail to show a significant difference between cases and controls ($\chi^2 = 2.9$, $P = 0.09$) but duration of use of OC is significant ($\chi^2 = 11.3$, $P < 0.001$).

### *Smoking*

Smoking was reported very rarely in this group of women and smoking habits did not differentiate cases from controls. Only nine cases and 12 controls were current smokers and seven cases against five controls were ex-smokers. The age- and race-adjusted odds ratio for ever smokers was 0.77 (95% CI = 0.27–2.12, $P = 0.65$).

### *Multivariate analysis*

In order to estimate the joint effect of the variables identified as potential risk factors, a selection of logistic regression

**Table II** Demographic characteristics

| | | *Cases* | | *Controls* | | *Crude odds ratio* | |
|---|---|---|---|---|---|---|---|
| *Variable* | *Levels* | *n* | *(%)* | *n* | *(%)* | *Point estimate* | *95% c.i.* |
| Place of birth | Singapore | 92 | (68) | 95 | (70) | 1[a] | – |
| | other | 42 | (31) | 40 | (30) | 1.08 | 0.64–1.82 |
| | n.k. | 1 | (1) | 0 | (–) | | |
| Accommodation | public | 115 | (85) | 125 | (93) | 1[a] | – |
| | private | 14 | (10) | 6 | (5) | 2.54 | 0.94–6.82 |
| | n.k. | 6 | (4) | 4 | (3) | 1.63 | 0.45–5.93 |
| Religion | Buddhist | 87 | (64) | 91 | (67) | 1[a] | – |
| | Muslim | 12 | (9) | 14 | (10) | 0.90 | 0.39–2.05 |
| | Christian | 5 | (4) | 9 | (7) | 0.58 | 0.19–1.80 |
| | Free Thinker | 4 | (3) | 9 | (7) | 0.47 | 0.14–1.57 |
| | Roman Cat. | 3 | (2) | 3 | (2) | 1.05 | 0.21–5.32 |
| | Hindu | 3 | (2) | 1 | (1) | 3.14 | 0.32–30.8 |
| | Ancestral | 9 | (7) | 0 | (–) | – | – |
| | Tao | 0 | (–) | 1 | (1) | – | – |
| | n.k. | 12 | (9) | 7 | (5) | 1.79 | 0.68–4.77 |
| Density (person/room) | (0, 1] | 32 | (24) | 44 | (32) | 1[a] | – |
| | (1, 2] | 77 | (57) | 77 | (57) | 1.38 | 0.79–2.39 |
| | (2, 3] | 17 | (13) | 9 | (7) | 2.60 | 1.03–6.57 |
| | >3 | 9 | (7) | 5 | (4) | 2.48 | 0.76–8.09 |
| | $\chi_1^2$ (trend) = 5.7 | | | | | | |
| Education | none | 53 | (39) | 36 | (27) | 1[a] | – |
| | primary | 50 | (37) | 56 | (42) | 0.61 | 0.34–1.07 |
| | secondary | 21 | (16) | 28 | (21) | 0.51 | 0.25–1.03 |
| | 'O' level or more | 5 | (4) | 14 | (10) | 0.24 | 0.08–0.74 |
| | n.k. | 6 | (4) | 1 | (1) | 4.08 | 0.47–35.3 |
| | $\chi_1^2$ (trend) = 13.3 | | | | | | |

[a]Reference group; n.k. – not known.

**Table III** Sexual and reproductive characteristics

| Variable | Levels | Cases n | (%) | Controls n | (%) | Crude odds ratio Point estimate | 95% c.i. |
|---|---|---|---|---|---|---|---|
| Age at menarche | ≤11 | 7 | (5) | 3 | (2) | 2.92 | 0.70–12.2 |
| | 12 | 26 | (19) | 21 | (16) | 1.55 | 0.74–3.24 |
| | 13 | 39 | (29) | 46 | (34) | 1.06 | 0.56–1.99 |
| | 14 | 31 | (23) | 25 | (19) | 1.55 | 0.77–3.13 |
| | ≥15 | 32 | (24) | 40 | (30) | 1[a] | – |
| | $\chi_1^2$ (trend) = 1.76, n.s. | | | | | | |
| Age at 1st intercourse | ≤17 | 38 | (28) | 19 | (14) | 3.90 | 1.76–8.65 |
| | 18–20 | 45 | (33) | 32 | (24) | 2.74 | 1.34–5.60 |
| | 21–24 | 33 | (24) | 36 | (27) | 1.79 | 0.86–3.70 |
| | ≥25 | 19 | (14) | 37 | (27) | 1[a] | – |
| | never | 0 | (–) | 11 | (8) | – | – |
| | $\chi_1^2$ (trend) = 28.99, $P<0.001$ | | | | | | |
| Parity | 0 | 1 | (1) | 25 | (19) | 0.04 | 0.01–0.33 |
| | 1 | 6 | (4) | 8 | (6) | 0.80 | 0.25–2.55 |
| | 2 | 32 | (24) | 34 | (25) | 1[a] | – |
| | 3 | 32 | (24) | 22 | (16) | 1.55 | 0.75–3.20 |
| | 4–5 | 30 | (22) | 26 | (19) | 1.23 | 0.60–2.50 |
| | ≥6 | 34 | (25) | 20 | (15) | 1.81 | 0.87–3.76 |
| | $\chi_1^2$ (trend) = 27.88, $P<0.001$ | | | | | | |
| Number of partners | 0 | 0 | (0) | 11 | (8) | – | – |
| | 1 | 110 | (82) | 116 | (86) | 1[a] | – |
| | ≥2 | 23 | (17) | 7 | (5) | 3.47 | 1.43–8.40 |
| | n.k. | 2 | (1) | 1 | (1) | 2.11 | 0.19–23.6 |
| | $\chi_1^2$ = 8.32[b], $P<0.001$ | | | | | | |
| Number of wives (of husbands) | 0 | 0 | (0) | 11 | (8) | – | – |
| | 1 | 108 | (80) | 109 | (81) | 1[a] | – |
| | ≥2 | 16 | (12) | 10 | (7) | 1.79 | 0.76–4.24 |
| | n.k. | 11 | (8) | 5 | (4) | 2.22 | 0.73–6.75 |
| | $\chi_1^2$ = 1.29[b], n.s. | | | | | | |

[a]Reference group. [b]For 1 vs 2 or more; n.k. – not known; n.s. – not significant

**Table IV** Correlation between risk factors in the control group excluding virgins (values in parentheses are for Chinese controls)

| | Education | Parity | Age at 1st intercourse |
|---|---|---|---|
| Parity | −0.41 (−0.39) | – | – |
| Age at 1st intercourse | 0.45 (0.41) | −0.50 (−0.46) | – |
| No. of partners | −0.11 (−0.08) | 0.01 (−0.05) | −0.19 (−0.16) |

models were fitted with the age and race matching properly taken into consideration (Breslow & Day, 1980).

Education, defined by the number of years in school, housing density and parity were all treated as linear variables. Use of barrier methods and use of oral contraceptives were defined as binary variables. Because of the small frequency of the extreme categories, number of partners was dichotomised to compare values greater than or equal to 2 against 0 or 1. Age at first intercourse (AFI) was also taken as a binary variable comparing age less than or equal to 20 against greater than 20 years. Virgins were

**Table V** Odds ratio for ever use of contraceptive methods (excluding virgins)

| Method | | Cases n | (%) | Controls n | (%) | Odds ratio and 95% c.i. (a) | (b) |
|---|---|---|---|---|---|---|---|
| Barrier (years) | never | 91 | (67) | 71 | (57) | 1[a] | 1[a] |
| | (0, 1] | 10 | (7) | 5 | (4) | 1.42(0.43–4.71) | 1.05(0.29–3.74) |
| | (1, 3] | 8 | (6) | 10 | (8) | 0.52(0.18–1.51) | 0.48(0.15–1.49) |
| | (3, 5] | 4 | (3) | 6 | (5) | 0.45(0.11–1.82) | 0.38(0.08–1.82) |
| | 5+ | 22 | (16) | 32 | (26) | 0.46(0.23–0.92) | 0.59(0.27–1.26) |
| | ever | 44 | (33) | 53 | (43) | 0.55(0.30–1.00) | 0.58(0.30–1.09) |
| | $\chi_1^2$ (trend) | | | | | 6.3 | 2.9 |
| OC (years) | never | 75 | (56) | 89 | (72) | 1[a] | 1[a] |
| | (0, 1] | 8 | (6) | 14 | (11) | 0.71(0.27–1.90) | 0.53(0.19–1.54) |
| | (1, 3] | 14 | (10) | 6 | (5) | 2.97(0.97–8.33) | 2.59(0.84–7.93) |
| | (3, 5] | 8 | (6) | 7 | (6) | 1.57(0.49–4.53) | 0.88(0.32–4.02) |
| | 5+ | 30 | (22) | 8 | (7) | 5.70(0.46–14.9) | 5.64(0.23–15.6) |
| | ever | 60 | (44) | 35 | (28) | 2.41(1.30–4.48) | 1.92(0.99–3.71) |
| | $\chi_1^2$ (trend) | | | | | 16.1 | 11.3 |
| IUD | never | 120 | (89) | 114 | (92) | 1[a] | 1[a] |
| | ever[b] | 15 | (11) | 10 | (8) | 1.39(0.58–3.35) | 1.36(0.52–3.96) |

[a]Reference group. [b]Ever users were too few for trend analysis; (a) adjusted for age, race and previous smear; (b) adjusted for age, race, previous smear, age at first intercourse, number of partners and education.

**Table VI** Summary of the multivariate analysis

| Model | Variable | Coefficient | (s.e.) | LR $\chi^2$ (1 df) | Deviance |
|---|---|---|---|---|---|
| A.1 | Parity (number) | 0.38 | (0.08) | 30.8 | 340.3 |
| A.2 | AFI ⩽20 | 1.20 | (0.28) | 19.0 | 352.1 |
| A.3 | ⩾2 partners | 1.08 | (0.36) | 17.1 | 354.0 |
| A.4 | Education (years) | −0.15 | (0.04) | 16.8 | 354.3 |
| B.1 | Parity | 0.32 | (0.08) | 19.2 | 332.9 |
| | AFI ⩽20 | 0.82 | (0.30) | 7.4 | – |
| B.2 | Parity | 0.38 | (0.08) | 29.4 | 324.6 |
| | ⩾2 partners | 1.03 | (0.36) | 15.8 | – |
| B.3 | Parity | 0.32 | (0.08) | 19.7 | 334.6 |
| | Education | −0.09 | (0.04) | 5.7 | – |
| B.4 | AFI ⩽20 | 1.01 | (0.29) | 13.8 | 341.2 |
| | ⩾2 partners | 0.91 | (0.36) | 10.9 | – |
| C.1 | Parity | 0.33 | (0.08) | 20.4 | 320.8 |
| | ⩾2 partners | 0.95 | (0.36) | 12.1 | – |
| | AFI ⩽20 | 0.60 | (0.31) | 3.5 | – |
| C.2 | Parity | 0.33 | (0.08) | 20.2 | 320.0 |
| | ⩾2 partners | 0.99 | (0.36) | 14.6 | – |
| | Education | −0.09 | (0.04) | 4.6 | – |

The analysis is standardised for the matching variables age and race.

included in the latter group. Other polytomous definitions of this variable were examined and found to give similar results and are not reported.

Table VI summarises the results obtained from alternative models for the data. Part A shows the estimated coefficients associated with parity, number of partners, age at first intercourse and education when these were considered separately. Parity emerges as the most predictive of the individual risk factors, although all four variables are highly significant. The most informative multivariate models are shown in parts B and C. Parity and number of partners appear to be independent variables and together appear to fit the data almost as well as more complicated models. Both of these are negatively correlated with AFI, the coefficient of which is much reduced when either of them are also included in the model. The estimated effect of number of partners could be overly influenced by the presence of 11 controls who had never had a sexual partner. When these were excluded from the analysis, no material difference was seen.

Specifications with other variables or combination of variables gave less significant or less parsimonious fits to the data and are not reported here. In particular, interaction terms were considered but were found to be non-significant.

### *Comparison of invasive and pre-invasive cases*

*Risk factors* Comparison of invasive and pre-invasive cases is examined in Table VII for a selection of variables. Some important differences are found: more of the pre-invasive cases than invasive cases were born in Singapore. They are also more educated, and a smaller proportion of them had more than three children. All these differences can be explained by the age distribution of the two groups: the mean age of the pre-invasive cases is 39.5 while that of the invasive cases is 51.4 (standard deviations, 7.3 and 13.0 respectively). Also, pre-invasive disease is almost always picked up at screening, which is more widely used by the upper socio-economic classes, and this could partly explain the differences in education and parity.

*Human papilloma virus (HPV)* The results of the analysis of biopsy material for HPV DNA are shown in Table VIII. Biopsies were collected for only nine pre-invasive cases (14.5%) and 29 invasive cases (39.7%). Of these 4/9 (44.4%) and 10/29 (34.5%), respectively, were found to have evidence of human papilloma virus DNA on Southern blot analysis. Of the 14 positives, 11 hybridised with HPV16, two with HPV31 and one with HPV6.

**Table VII** Characteristics of pre-invasive and invasive cases

| Variable | Levels | Pre-invasive n | (%) | Invasive n | (%) |
|---|---|---|---|---|---|
| Age (years) | ⩽30 | 6 | (10) | 4 | (5) |
| | 31–40 | 31 | (50) | 12 | (16) |
| | 41–50 | 20 | (32) | 22 | (30) |
| | 51–60 | 5 | (8) | 16 | (22) |
| | >60 | 0 | (–) | 19 | (26) |
| Mean | | 39.5 | | 51.4 | |
| s.d. | | 7.3 | | 13.0 | |
| Race | Chinese | 58 | (94) | 61 | (84) |
| | Indian | 1 | (2) | 2 | (3) |
| | Malay | 3 | (5) | 10 | (14) |
| Place of birth | Singapore | 53 | (86) | 39 | (53) |
| | Other | 9 | (15) | 33 | (45) |
| | n.k. | 0 | (–) | 1 | (1) |
| Education | None | 14 | (23) | 39 | (53) |
| | Primary | 27 | (44) | 23 | (32) |
| | Secondary | 16 | (26) | 5 | (7) |
| | 'O' level or more | 3 | (5) | 2 | (3) |
| | n.k. | 2 | (3) | 4 | (6) |
| Age at 1st intercourse | ⩽17 | 9 | (15) | 29 | (40) |
| | 18–20 | 19 | (31) | 26 | (36) |
| | 21–24 | 20 | (32) | 13 | (18) |
| | ⩾25 | 14 | (23) | 5 | (7) |
| | never | 0 | (–) | 0 | (–) |
| Parity | 0 | 1 | (2) | 0 | (–) |
| | 1 | 5 | (8) | 1 | (1) |
| | 2 | 21 | (34) | 11 | (15) |
| | 3 | 20 | (32) | 12 | (17) |
| | 4–5 | 7 | (11) | 23 | (32) |
| | ⩾6 | 8 | (13) | 26 | (36) |
| No. of partners | 0 | 0 | (–) | 0 | (–) |
| | 1 | 56 | (90) | 54 | (74) |
| | ⩾2 | 6 | (10) | 17 | (23) |
| | n.k. | 0 | (–) | 2 | (2) |

n.k. – not known.

**Table VIII** HPV hybridisation results

| Results | | Pre-invasive | | Invasive | | Total | |
|---|---|---|---|---|---|---|---|
| Negative | | 5 | (55.6%) | 19 | (65.5%) | 24 | (63.2%) |
| Positive | HPV6 | 1 | (44.4%) | 0 | (34.5%) | 1 | (36.8%) |
| | HPV16 | 3 | | 8 | | 11 | |
| | HPV31 | 0 | | 2 | | 2 | |
| Total | | 9 | (100.0%) | 29 | (100.0%) | 38 | (100.0%) |
| Biopsy not available | | 53 | | 44 | | 97 | |

### Discussion

Parity, age at first intercourse and number of partners all emerge as important prognostic factors in this study. Unfortunately they are highly correlated and so it is difficult to determine which is most important or even which factors produce independent risks. In our data parity and number of partners appeared most independent and, after adjusting for them, age at first intercourse was only marginally significant ($P=0.05$).

This result differs from Peters *et al.* (1986), who found that parity lost significance when number of partners and age at first intercourse were included in the model, and from Brinton *et al.* (1987), who found that number of partners and age at first intercourse were independent factors in a study of US urban women. A possible explanation for the difference might be the confounding effect of unrecorded sexual behaviour of male partners, which is possibly more relevant in this part of the world than in the USA. The women in our study had fewer reported sexual partners than in similar studies in Western countries and it is possible that promiscuous behaviour by males has a greater impact here than in the West (Skegg *et al.*, 1982). Male sexual behaviour is broadly recognised to play an important role in the woman's exposure to causative agent(s) for cervix cancer (Buckley *et al.*, 1981; Campion *et al.*, 1988) and in our study is only weakly measured by the number of previous wives of the current/last husband. The relative risk for age at first intercourse and number of partners estimated in this study are nevertheless in general agreement with those found in a Thai population (Wanguphachart *et al.*, 1987) and in studies on Western populations. Brinton *et al.* (1987) found relative risks for age at first intercourse less or equal 20 or for two or more sexual partners of just over 2, whereas our (adjusted) values are 1.8 and 2.6, respectively. Other studies found similar (La Vecchia *et al.*, 1986) or moderately larger (Peters *et al.*, 1986) values.

Recent studies have reported raised risks of invasive cervical cancer for long-term users of oral contraceptives (WHO, 1985; Brinton *et al.*, 1986). These findings were adjusted for sexual activity and screening history, while previous studies reporting contrasting results could not adjust for these factors. In our data the raised relative risk for oral contraceptives users lost significance when adjusted for sexual activity, screening history and education but the trend test for duration of use was significant. Use of barrier methods also lost significance when the adjusted odds ratio was computed but did not show a significant trend for duration of use.

Of the factors not directly related to sexual behaviour, education was the most significant, in agreement with a similar study on Thai women (Wanguphachart *et al.*, 1987) and with a study on Hispanics and non-Hispanics in California (Peters *et al.*, 1986). It is unclear how education affects risks, but it could be through diet, hygiene or sexual behaviour. Smoking is not a very common habit among women in Singapore, except for older Cantonese women MacLennan *et al.*, 1977), and the overall prevalence in our study was only 12%. The prevalence of HPV DNA of types 16, 18 and 31 was lower in this study (37%) than in many Western series, where types 16 or 18 are found in 40–90% of invasive cancers (zur Hausen, 1984; Lorincz *et al.*, 1987; Muñoz *et al.*, 1988). The reasons for an apparent lower prevalence of HPV infection in a region of high incidence of cervix cancer requires further studies.

We thank the doctors of the Bedok, Kelantin and Bukit Merah Clinics for providing controls, Mrs Jane Webster and Mrs Anny Leow for interviewing, and gratefully acknowledge a supporting grant from the Singapore Cancer Society.

### References

BARRASSO, R., DE BRUX, J., CROISSANT, O. & ORTH, G. (1987). High prevalence of papillomavirus-associated penile intra-epithelial neoplasia in sexual partners of women with cervical intraepithelial neoplasia. *N. Engl. J. Med.*, **317**, 916.

BERAL, V. (1974). Cancer of the cervix: a sexually transmitted disease. *Lancet*, **ii**, 1037.

BOYD, J.T. & DOLL, R. (1964). A study of the aetiology of carcinoma of the cervix uteri. *Br. J. Cancer*, **18**, 419.

BRESLOW, N.E. & DAY, N.E. (1980). *Statistical Methods in Cancer Research.* IARC: Lyon.

BRINTON, L.A., HUGGINS, G.R., LEHMAN, H.F., MALLIN, K. and 5 others (1986). Long-term use of oral contraceptives and risk of invasive cervical cancer. *Int. J. Cancer*, **38**, 339.

BRINTON, L.A., HAMMAN, R.F., HUGGINS, G.R. and 4 others (1987). Sexual and reproductive risk factors for invasive squamous cell cervical cancer. *J. Natl Cancer Inst.*, **79**, 23.

BUCKLEY, J.D., HARRIS, R.W.C., DOLL, R., VESSEY, M.P. & WILLIAMS, P.T. (1981). Case–control study of the husbands of women with dysplasia or carcinoma of the cervix uteri. *Lancet*, **ii**, 1010.

CAMPION, M.J., SINGER, A., CLARKSON, P.K. & McCANCE, D.J. (1985). Increased risk of cervical neoplasia in consorts of men with penile condylomata acuminata. *Lancet*, **i**, 943.

CAMPION, M.J., McCANCE, D.J., CUZICK, J. & SINGER, A. (1986). The progressive potential of mild cervical atypia: a colposcopic, cytological and virological study. *Lancet*, **ii**, 237.

CRAMER, D.W. (1982). Uterine cervix. In *Cancer Epidemiology and Prevention*, Schottenfeld, D. & Fraumeni, J.F. (eds). W.B. Saunders: Philadelphia.

DE VILLIERS, E.-M., SCHNEIDER, A., MIKLAW, H. and 5 others (1987). Human papillomavirus infections in women with and without abnormal cervical cytology. *Lancet*, **ii**, 703.

DURST, M., GISSMANN, L., IKENBERG, H. & ZUR HAUSEN, H. (1983). A papillomavirus DNA from a cervical carcinoma and its prevalence in cancer biopsy samples from different geographic regions. *Proc. Natl Acad. Sci. USA*, **80**, 3812.

FRANCESCHI, S., DOLL, R., GALLWEY, J., LA VECCHIA, C., PETO, R. & SPRIGGS, A.I. (1983). Genital warts and cervical neoplasia: an epidemiological study. *Br. J. Cancer*, **48**, 621.

GRAHAM, S. & SCHOTZ, W. (1979). Epidemiology of cancer of the cervix in Buffalo, New York. *J. Natl Cancer Inst.*, **63**, 23.

GRUBB, G.S. (1986). Human papillomavirus and cervical neoplasia: epidemiological considerations. *Int. J. Epidemiol.*, **15**, 1.

HARRIS, R.W.C., BRINTON, L.A., COWDELL, R.H. and 4 others (1980). Characteristics of women with dysplasia or carcinoma *in situ* of the cervix uteri. *Br. J. Cancer*, **42**, 359.

HARRIS, R.W.C., FORMAN, D., DOLL, R., VESSEY, M.P. & WALD, N.J. (1986). Cancer of the cervix uteri and vitamin A. *Br. J. Cancer*, **53**, 653.

JUSSAWALLA, D.J., DESHPANDE, V.A. & STANDFAST, S.J. (1971). Assessment of risk patterns in cancer of the cervix. A comparison between Greater Bombay and western countries. *Int. J. Cancer*, **7**, 259–268.

KESSLER, I. (1976). Human cervical cancer as a veneral disease. *Cancer Res.*, **36**, 783.

LA VECCHIA, C., FRANCESCHI, S., DECARLI, A. and 4 others (1986). Sexual factors, venereal diseases and the risk of intra-epithelial and invasive cancer neoplasia. *Cancer*, **58**, 935.

LORINCZ, A.T., TEMPLE, G.F., KURMAN, R.J., BENNETT JENSON, A. & LANCASTER, W.D. (1987). Oncogenic association of specific human papillomavirus types with cervical neoplasia. *J. Natl Cancer Inst.*, **79**, 671.

MacLENNAN, R., DA COSTA, J., DAY, N.E., LAW, C.H., NG, Y.K. & SHANMUGARATNAM, K. (1977). Risk factors for lung cancer in Singapore Chinese, a population with high female incidence rates. *Int. J. Cancer*, **20**, 854.

MARSHALL, J.R., GRAHAM, S., BYERS, T., SWANSON, M. & BRASURE, J. (1983). Diet and smoking in the epidemiology of cancer of the cervix. *J. Natl Cancer Inst.*, **70**, 847.

MARTIN, C.E. (1967). Marital and coital factors in cervical cancer. *Am. J. Pub. Health*, **57**, 803.

MUÑOZ, N., BOSCH, X. & KALDOR, J.M. (1988). Does human papillomavirus cause cervical cancer? The state of the epidemiological evidence. *Br. J. Cancer*, **57**, 1.

McCANCE, D.J., CAMPION, M.J., CLARKSON, P.K., CHESTERS, P.M., JENKINS, D. & SINGER, A. (1985). Prevalence of human papillomavirus type 16 DNA sequences in cervical intraepithelial neoplasia and invasive carcinoma of the cervix. *Br. J. Obstet. Gynaecol.*, **92**, 1101.

PETERS, R.K., THOMAS, D., HAGAN, D.G., MACK, T.M. & HENDERSON, B.E. (1986). Risk factors for invasive cervical cancer among Latinas and non-Latinas in Los Angeles County. *J. Natl Cancer Inst.*, **77**, 1063.

RAWLS, W.E., LAVERY, C., MARRETT, L.D. and 9 others (1986). Comparison of risk factors for cervical cancer in different populations. *Int. J. Cancer*, **37**, 537.

ROTKIN, I.D. (1967). Adolescent coitus and cervical cancer: associations of related events with increased risk. *Cancer Res.*, **27**, 603.

ROTKIN, I.D. (1973). A comparison review of key epidemiological studies in cervical cancer related to current searches for transmissible agents. *Cancer Res.*, **33**, 1353.

SKEGG, D.C., CORWIN, D.A., PAUL, C. & DOLL, R. (1982). Importance of the male factor in cancer of the cervix. *Lancet*, **ii**, 581.

SMITH, P.G., KINLEN, L.J., WHITE, G.C., ADELSTEIN, A.M. & FOX, A.J. (1980). Mortality of wives of men dying with cancer of the penis. *Br. J. Cancer*, **41**, 422.

VESSEY, M.P., LAWLESS, M., McPHERSON, K. & YEATES, D. (1983). Neoplasia of the cervix uteri and contraception: a possible adverse effect of the pill. *Lancet*, **ii**, 930.

VILLA, L.L. & LOPES, A. (1986). Human papillomavirus DNA sequences in penile carcinomas in Brazil. *Int. J. Cancer*, **37**, 853.

WALKER, P., SINGER, A., DYSON, J. & ORIEL, D. (1984). The natural history of cervical epithelial abnormalities in patients with vulval warts. *Br. J. Vener. Dis.*, **59**, 327.

WANGSUPHACHART, V., THOMAS, D.B., KOETSAWANG, A. & RIOTTON, G. (1987). Risk factors for invasive cervical cancer and reduction of risk by 'Pap' smears in Thai women. *Int. J. Epidemiol.*, **16**, 362.

WINKELSTEIN JR, W., SHILLITOE, E.J., BRAND, R. & JOHNSON, K.K. (1984). Further comments on cancer of the uterine cervix, smoking, and herpesvirus infection. *Am. J. Epidemiol.*, **119**, 1.

WHO (1985). Collaborative Study of Neoplasia and Steroid Contraceptives. Invasive cervical cancer and combined oral contraceptives. *Br. Med. J.*, **290**, 961.

WYNDER, E.L., CORNFIELD, J., SCHNAFF, P.D. & DORAISWAMI, K.R. (1954). A study of environmental factors in cancer of the cervix. *Am. J. Obstet. Gynecol.*, **68**, 1016.

ZUNZUNEGUI, M.V., KING, M.-C., CORIA, C.F. & CHARLET, J. (1986). Male influences on cervical cancer risk. *Am. J. Epidemiol.*, **123**, 302.

ZUR HAUSEN, H. (1984). Viruses in the aetiology of human genital cancer. *Prog. Med. Virol.*, **30**, 170.